# Blood–brain barrier transcytosis genes, risk of dementia and stroke: a prospective cohort study of 74,754 individuals

**DOI:** 10.1007/s10654-019-00498-2

**Published:** 2019-03-04

**Authors:** Ida Juul Rasmussen, Anne Tybjærg-Hansen, Katrine Laura Rasmussen, Børge G. Nordestgaard, Ruth Frikke-Schmidt

**Affiliations:** 1 Department of Clinical Biochemistry KB 3011, Rigshospitalet, Copenhagen University Hospital, Blegdamsvej 9, 2100 Copenhagen Ø, Denmark; 2 The Copenhagen General Population Study, Herlev and Gentofte Hospital, Herlev Ringvej 75, 2730 Herlev, Denmark; 3 The Copenhagen City Heart Study, Frederiksberg Hospital, 2000 Frederiksberg, Denmark; 4 Department of Clinical Medicine, Faculty of Health and Medical Sciences, University of Copenhagen, Blegdamsvej 3, 2200 Copenhagen, Denmark; 5 Department of Clinical Biochemistry, Herlev and Gentofte Hospital, Herlev Ringvej 75, 2730 Herlev, Denmark

**Keywords:** Alzheimer’s disease, Vascular dementia, Amyloid-β, Epidemiology, Stroke, Blood–brain barrier clearance

## Abstract

To test whether genetic variants in *PICALM*, *BIN1*, *CD2AP*, and *RIN3*—suggested to be involved in blood–brain barrier amyloid-β transcytosis pathways—associate with Alzheimer’s disease, all dementia, suggested vascular dementia, and stroke, and whether such associations are independent of the strong ε4 *APOE* risk allele. In a prospective cohort study of 74,754 individuals from the general population we genotyped *PICALM* (rs10792832), *BIN1* (rs6733839), *CD2AP* (rs10948363), and *RIN3* (rs10498633), and generated a weighted and a simple allele score. Multifactorially adjusted hazard ratios for the fourth quartile versus the first quartile of the weighted allele score were 1.42 (95% confidence interval 1.22–1.64) for Alzheimer’s disease, and 1.33 (1.19–1.48) for all dementia. For suggested vascular dementia and stroke the corresponding estimates were 1.71 (1.18–2.49) and 1.12 (1.04–1.22), respectively. Hazard ratios were similar after *APOE* adjustment. Genetic variants in *PICALM*, *BIN1*, *CD2AP*, and *RIN3* are associated with increased risk of Alzheimer’s disease, all dementia, and suggested vascular dementia independent of the strong *APOE* ε4 allele. These findings may suggest that clathrin-mediated endocytosis in clearance of amyloid-β across the blood–brain barrier is important for the integrity of both brain tissue and cerebral vessels.

**Electronic supplementary material:**

The online version of this article (10.1007/s10654-019-00498-2) contains supplementary material, which is available to authorized users.

## Introduction

Alzheimer’s disease and other forms of dementia are devastating neurodegenerative diseases currently affecting more than 47 million people globally, and expected to triple in 2050 [1]. There are no available curative treatments, no early preclinical, easily accessible biomarkers [2, 3], and large parts of the underlying biology remain unknown. Clinically, Alzheimer’s disease often coexists with cerebral vascular diseases [4–8], and the major pathological hallmark of Alzheimer’s disease is accumulation of a neurotoxic, sticky peptide, amyloid-β, in the brain and in cerebral vessels [3, 9–12]. An important clearance pathway of amyloid-β from the brain is via transcytosis across the blood–brain barrier into the vascular lumen [13–15].

Interestingly, a number of genome-wide association studies (GWAS) risk genes [16], *PICALM* (phosphatidylinositol-binding clathrin assembly protein) [17, 18], *BIN1* (bridging integrator 1) [19–21], *CD2AP* (CD2-associated protein) [22, 23], and *RIN3* (Ras and Rab interactor 3) [24], encode proteins that are either directly or indirectly involved in transcytosis of amyloid-β across the blood–brain barrier [24–32]. Particularly genetic variants tagging *PICALM* consistently associate with dementia in GWAS [16, 30, 33–35], and this signal is one of the most consistent after the ε4 allele of *APOE* (apolipoprotein E gene) [29], an association first recognized in 1993 [36] and since validated globally [2, 3, 16, 30, 31, 35, 37]. PICALM is important for endocytosis and internalization of cell receptors, and is involved in clathrin-mediated endocytosis [28, 38]. Recently it was shown that PICALM regulates amyloid-β blood–brain barrier transcytosis and clearance by initiating clathrin-mediated endocytosis via interaction with LRP1 (low-density lipoprotein receptor related protein-1). LRP1 is a key amyloid-β clearance acceptor that also binds to apoE [39].

Our aim was dual. First, we wanted to establish the exact risk increases at the individual level for Alzheimer’s disease and all dementia for four potential blood–brain barrier pathway genes. Second, we wanted to test the impact on specific cerebral vascular endpoints –suggested vascular dementia and stroke—since the blood–brain barrier most likely is central for dementia related vascular events. We genotyped four variants in *PICALM*, *BIN1*, *CD2AP*, and *RIN3* previously identified as top hits for Alzheimer’s disease [16], in two prospective studies of the general population totaling 74,754 individuals.

## Materials and methods

### Participants

The Copenhagen General Population Study (CGPS) is a prospective study of the Danish general population initiated in 2003 and still recruiting [40–42]. Individuals were selected randomly based on the national Danish Civil Registration System to reflect the adult Danish population aged 20–80 + years. Data were obtained from a self-administered questionnaire reviewed together with an investigator at the day of attendance, a physical examination, and from blood samples including DNA extraction. Genotypes are available on 64,974 individuals. Before genotyping all DNA samples were blinded regarding phenotype and endpoint to the laboratory technician and investigator.

The Copenhagen City Heart Study (CCHS) is a prospective study of the Danish general population initiated in 1976–1978 with follow-up examinations in 1981–1983, 1991–1994 and 2001–2003 [40–42]. Individuals were recruited and examined exactly as in the CGPS. Genotypes are available on 9780 individuals from the 1991–1994 and 2001–2003 examinations.

Combining the two studies yielded a total of 74,754 individuals for Alzheimer’s disease and all dementia analyses, of whom 2514 developed dementia during a median follow-up of 10 years (range 0–23 years). For the analyses of suggested vascular dementia and stroke we included 72,612 individuals, of whom 5016 developed stroke and 248 developed suggested vascular dementia during a median follow-up of 10 years (range 0–25 years). No individuals were lost to follow-up. Follow-up began at the time of blood sampling (2003 and onward for CGPS and 1991–1994 or 2001–2003 for CCHS). Follow-up ended at occurrence of event (n = 2514 for Alzheimer’s disease and all dementia and n = 5264 for suggested vascular dementia and stroke), death (n = 10,608 for Alzheimer’s disease and all dementia and n = 9203 for suggested vascular dementia and stroke), emigration (n = 404 for Alzheimer’s disease and all dementia and n = 395 for suggested vascular dementia and stroke), or on March 22nd, 2017 (last update of the registry), whichever came first. Years at risk were calculated for each participant as the time difference between baseline and the end of follow-up. Written informed consent was obtained from all individuals. Individuals in both studies were white and of Danish descent.

### Dementia and stroke

In CGPS and CCHS, information on births, deaths, emigrations and immigrations was collected from the national Danish Civil Registration System. Information on diagnoses of dementia and stroke, and age at diagnosis was drawn from the national Danish Patient Registry and the national Danish Causes of Death Registry. The national Danish Patient Registry has information on all patient contacts with all clinical hospital departments in Denmark since 1977, including emergency wards and outpatient clinics from 1994. The national Danish Causes of Death Registry contains data on the causes of all deaths in Denmark, as reported by hospitals and general practitioners. The national Danish registries are regarded among the best of its kind [43, 44], however an inherent limitation of using registry-based data is underdiagnosis, because only hospital registered events are in the registries. This is a general issue that needs to be considered when interpreting results based on registry diagnoses. In Denmark the diagnosis of dementia has been made in accordance with international standards in routine clinical practice since the 1990’s and 91–95% of diagnoses are given by neurologically relevant units and/or departments of internal medicine [45]. Alzheimer’s disease is diagnosed using the NINCDS-ADRDA criteria [46], the NIA-AA criteria [47], or the ICD8/ICD10 criteria and is highly valid once the diagnosis is given [48]. Alzheimer’s disease was ICD8 (World Health Organization International Classification of Diseases, 8th revision) code 290.10 and ICD10 (World Health Organization International Classification of Diseases, 10th revision) codes F00 and G30. Vascular dementia has been diagnosed using the ICD10 criteria, the NINDS-AIREN criteria (before 2015) [49] or the VASCOG criteria since 2015 [50]. The diagnosis, however, suffer from some uncertainty [48], which is why we use the term “suggested vascular dementia” throughout the paper. The diagnosis of suggested vascular dementia was ICD10 code F01 and did not include mixed dementia. All dementia further included unspecified dementia (ICD8 290.18; ICD10 F03). Stroke was ICD8 codes 430, 431 and 433–435 and ICD10 code G45, I60, I61, I63 and I64.

### Laboratory analyses

Standard hospital assays measured electrolytes, glucose, liver-, kidney-, and inflammatory parameters as well as total cholesterol, HDL (high-density lipoprotein) cholesterol and triglycerides (Boehringer Mannheim, Mannheim, Germany). LDL (low-density lipoprotein) cholesterol was calculated using the Friedewald equation [51] when plasma triglycerides were ≤ 4 mmol/L (≤ 352 mg/dL), and otherwise measured directly (Konelab). Estimated glomerular filtration rate was calculated according to CKD-EPIcrea [52].

### Genotyping

Taqman-based (Life Technologies, a part of Thermo Fisher Scientific, Waltham, Massachusetts, USA) or KASP technology based assays (LGC Genomics, Hoddesdon, Herts, UK) were used to genotype for *PICALM* rs10792832, *BIN1* rs6733839, *CD2AP* rs10948363, *RIN3* rs10498633 and for p.Cys130Arg (rs429358, legacy name Cys112Arg, c.388T>C) defining the ε4 allele and p.Arg176Cys (rs7412, legacy name Arg158Cys, c.526C>T) defining the ε2 allele of the *APOE* gene [42].

### Other covariates

Body mass index was measured weight in kilograms divided by measured height in meters squared. Hypertension was use of anti-hypertensive medication, a systolic blood pressure of 140 mm Hg or greater, and/or a diastolic blood pressure of 90 mm Hg or greater. Diabetes mellitus was self-reported disease, use of insulin or oral hypoglycemic agents, and/or non-fasting plasma glucose levels of more than 11 mmol/L (> 198 mg/dL). Estimated glomerular filtration rate was calculated according to CKD-EPIcrea [52]. Smoking was current smoking. High alcohol consumption was > 14/21 units per week for women/men (1 unit = 12 g alcohol, equivalent to one glass of wine or one beer (33 cL)). Physical inactivity was ≤ 4 h per week of light physical activity in leisure time. Women reported menopausal status and use of hormonal replacement therapy. Lipid-lowering therapy was mainly statins (yes/no), and low education was < 8 years of education.

### Consortia data

IGAP (the International Genomics of Alzheimer’s Project) (is a large two-stage study based upon GWAS on individuals of European ancestry [16]. In stage 1, IGAP used genotyped and imputed data on 7,055,881 SNPs (single nucleotide polymorphisms) to meta-analyze four previously published GWAS datasets consisting of 17,008 Alzheimer’s disease cases and 37,154 controls (The European Alzheimer’s disease Initiative—EADI, the Alzheimer Disease Genetics Consortium—ADGC, the Cohorts for Heart and Aging Research in Genomic Epidemiology consortium—CHARGE, the Genetic and Environmental Risk in AD consortium—GERAD). In stage 2, 11,632 SNPs were genotyped and tested for association in an independent set of 8572 Alzheimer’s disease cases and 11,312 controls. Finally, a meta-analysis was performed combining results from stages 1 and 2 [16].

### Statistical analysis

We used Stata/S.E. v14.0 and v13.0 (Stata Corp, College Station, TX, USA). Probability values < 0.001 are given as powers of 10. Kruskal–Wallis one-way analysis of variance or Pearson’s *χ*^2^ test were used to evaluate continuous and categorical variables by genotype and disease status. Missing data on continuous covariates were imputed from age, sex, and the most related continuous parameters. Missing data on categorical covariates were assigned a dummy value. Missing values for continuous covariates were < 0.8%.

Combining all genotypes, we generated two different genetic risk scores for dementia and stroke. The first genetic score, named “weighted allele score”, was calculated for each individual using a weighted sum of alleles for increasing risk of Alzheimer’s disease and all dementia, subsequently categorized into quartiles of approximately equal size to maximize statistical power. The weights correspond to the regression coefficients for the dementia risk increasing alleles in each individual adjusted for age and gender [53] (Supplementary Table 1). The weights were generated in the combined cohort. The second genetic score, named “simple allele score”, was a simple counting of the number of dementia increasing risk alleles in each individual, subsequently categorized into three groups of approximately equal size. Similar scores were generated for suggested vascular dementia and stroke.

Cumulative incidences of Alzheimer’s disease and all dementia were plotted against age and weighted/simple allele score group, using the method of Fine-Gray [54], to account for the possibility of death as a competing event. Similar cause-specific (censoring at death) Cox proportional hazards regression models with age as time scale and left truncation (delayed entry) were used to estimate hazard ratios for Alzheimer’s disease, all dementia, suggested vascular dementia, and stroke as a function of weighted allele score and simple allele score. Using age as time scale ensures that each participant experiencing an event is always compared to a participant at the exact same age. This is regarded as the state of the art age-adjustment method in survival analyses in large epidemiological studies where participants enter at different ages. For Cox regression models, proportionality of hazards over time were assessed by plotting − ln(− ln[survival]) versus ln(analysis time). There was no suspicion of nonproportionality. Cox regression models were multifactorially adjusted for age (as time scale), sex, body mass index, hypertension, diabetes, smoking, alcohol intake, physical inactivity, postmenopausal status and hormonal replacement therapy in women, lipid-lowering therapy, educational level, and *APOE* genotype (ε2/ε3/ε4 *APOE* genotype).

Meta-analyses were conducted using the user-written metan command from Stata/S.E. v13.0 to estimate fixed and random effects odds ratios by regression coefficients and standard errors for each of the four genetic variants. Between-study heterogeneity was assessed by Cochran’s Q test and I^2^ statistics and was considered as low (I^2^ ≤ 25%), moderate (25% < I^2^ < 49%), or high (I^2^ ≥ 50%). Results are presented for both fixed- and random-effects models.

## Results

Baseline characteristics of the 74,754 individuals enrolled in the study are shown by quartiles of weighted allele score for Alzheimer’s disease in Table 1. Baseline characteristics by disease status are shown in Supplementary Table 2. Age at diagnosis of disease are shown in Supplementary Table 3 and distribution of age at baseline and follow-up time are shown in density plots in Supplementary Figures 1 and 2. All results are for the CGPS and CCHS combined unless otherwise stated.

**Table 1:** Characteristics of study participants by weighted allele score quartile for Alzheimer’s disease

	1st quartile	2nd quartile	3rd quartile	4th quartile
No. of individuals (%)	19,423 (26)	18,251 (24)	18,409 (25)	18,671 (25)
Age (years)	58 (48–67)	58 (48–67)	58 (47–67)	57 (47–67)
Female (%)	55	56	55	56
Total cholesterol (mmol/L)	5.6 (4.9–6.4)	5.6 (4.9–6.4)	5.6 (4.9–6.4)	5.6 (4.9–6.4)
LDL cholesterol (mmol/L)	3.3 (2.6–3.9)	3.2 (2.6–3.9)	3.2 (2.6–3.9)	3.3 (2.6–3.9)
HDL cholesterol (mmol/L)	1.5 (1.2–1.9)	1.6 (1.3–1.9)	1.5 (1.2–1.9)	1.6 (1.2–1.9)
Triglycerides (mmol/L)	1.4 (1.0–2.1)	1.4 (1.0–2.1)	1.4 (1.0–2.1)	1.4 (1.0–2.1)
Body mass index (kg/m^2^)	26 (23–28)	26 (23–28)	26 (23–28)	26 (23–28)
Hypertension (%)	58	59	58	58
Diabetes mellitus (%)	4	4	4	4
Smoking (%)	23	24	24	24
High alcohol consumption (%)	18	18	18	18
Physical inactivity (%)	53	52	53	52
Postmenopausal (%)^a^	66	66	67	67
Hormonal replacement therapy (%)^a^	11	12	11	11
Lipid-lowering therapy (%)	9	10	9	9
Education < 8 years (%)	14	14	14	15

Values are median (interquartile range) or percent and are from the day of enrolment (2003 and onwards for the CGPS and 1991–1994 or 2001–2003 for the CCHS). Hypertension was use of anti-hypertensive medication, a systolic blood pressure of 140 mm Hg or greater, and/or a diastolic blood pressure of 90 mm Hg or greater. Diabetes mellitus was self-reported disease, use of insulin or oral hypoglycemic agents, and/or non-fasting plasma glucose levels of more than 11 mmol/L (> 198 mg/dL). Smoking was current smoking. High alcohol consumption was > 14/21 units per week for women/men (1 unit = 12 g alcohol, equivalent to one glass of wine or one beer (33 cL)). Physical inactivity was ≤ 4 h per week of light physical activity in leisure time. Women reported menopausal status and use of hormonal replacement therapy. Lipid-lowering therapy was primarily statins (yes/no), and low education was < 8 years of education. Differences across weighted allele score groups were tested by Kruskal–Wallis one-way analysis of variance or Pearson’s *χ*^2^-test

*HDL* high-density lipoprotein cholesterol, *LDL* low-density lipoprotein cholesterol

^a^In women only

### Weighted/simple allele scores and risk of Alzheimer’s disease and all dementia

**Fig. 1:**
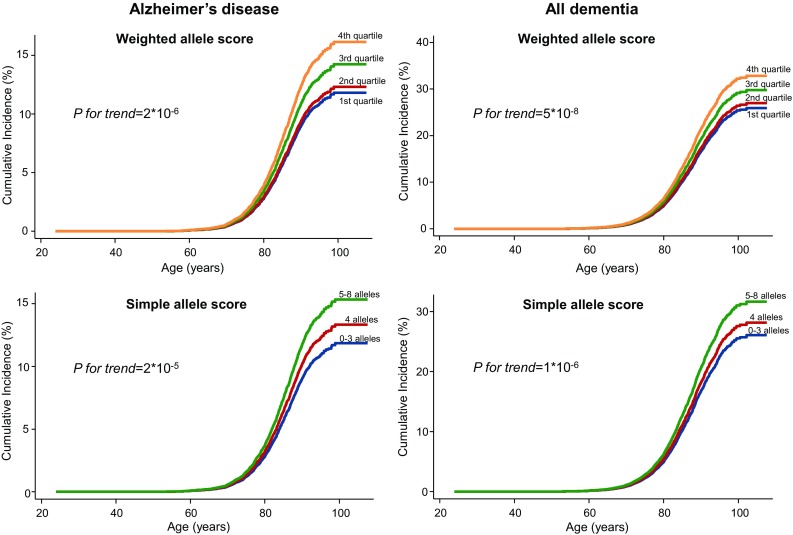
Cumulative incidence of Alzheimer’s disease and all dementia by age and weighted/simple allele scores. We used Fine-Gray models, allowing for death as a competing event. *P* for trend from competing risks regression trend test. Weights for the weighted allele score were generated in the combined cohort

**Fig. 2:**
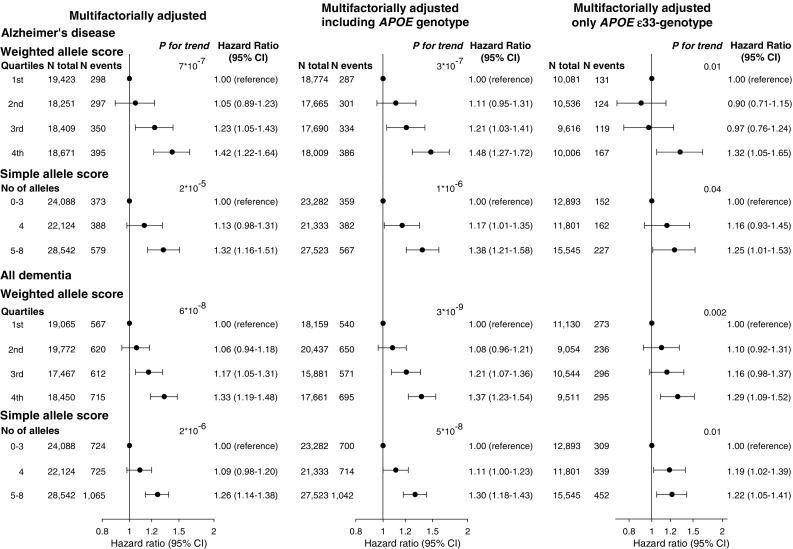
Risk of Alzheimer’s disease and all dementia as a function of weighted/simple allele scores. Individuals with Alzheimer’s disease or all dementia before blood sampling were excluded, leaving 74,754 individuals for analysis in the left panel. A total of 72,138 individuals with available *APOE* genotype were included in the middle panel. The right panel exclusively contains individuals with the *APOE* ε33 genotype (N = 40,239). Hazard ratios were multifactorially adjusted for age (as time scale), sex, hypertension, diabetes, smoking, alcohol intake, physical inactivity, postmenopausal status and hormonal replacement therapy in women, lipid-lowering therapy and educational level (left and right panel). Middle panel additionally includes adjustment for *APOE* genotype. *P* for trend from Cox regression. *APOE *= apolipoprotein E gene; *APOE* genotype = ε2/ε3/ε4 *APOE* genotype; CI = confidence interval

Cumulative incidences of Alzheimer’s disease and all dementia increased stepwise as a function of increasing weighted/simple allele score groups (all *P* for trend ≤ 2 × 10^−5^) (Fig. 1). Multifactorially adjusted hazard ratios for the fourth versus the first weighted allele score quartile were 1.42 (95% confidence interval 1.22–1.64) for Alzheimer’s disease and 1.33 (1.19–1.48) for all dementia. For the simple allele score multifactorially adjusted hazard ratios for 5–8 alleles versus 0–3 alleles were 1.32 (1.16–1.51) for Alzheimer’s disease and 1.26 (1.14–1.38) for all dementia (Fig. 2, left panel). Results were similar after further adjustment for *APOE* genotype (Fig. 2, middle panel), or when analyses were performed exclusively in *APOE* ε33 carriers (Fig. 2, right panel). Findings for Alzheimer’s disease were comparable to results using external weights (Supplementary Figure 3) [16]; external weights were not available for all dementia. Findings were similar for the CGPS and CCHS separately (Supplementary Figures 4 and 5) and when excluding participants who developed dementia of any subtype within the first six months of follow-up (Supplementary Figure 6). When dividing the weighted allele score into tertiles, hazard ratios for the third tertile were similar to hazard ratios for the fourth quartile in the original analysis for both Alzheimer’s disease and all dementia (Supplementary Figure 7). We also performed the analysis in *APOE* ε4-carriers and ε4-non-carriers separately and results were similar (Supplementary Figure 8). After stratification in age groups (< 65 years, 65–80 years and ≥ 80 years), results were similar although attenuated in the ≥ 80 years age group, most likely due to lack of power (Supplementary Figure 9). There was no interaction between the genetic scores and age at baseline (Alzheimer’s disease: *P *= 0.43 for the weighted allele score and *P *= 0.61 for the simple allele score. All dementia: *P *= 0.53 for the weighted allele score and *P *= 0.18 for the simple allele score). Further no interaction was present between the genetic scores and *APOE* genotype (Alzheimer’s disease: *P* = 0.31 for the weighted allele score and *P* = 0.52 for the simple allele score. All dementia: *P* = 0.91 for the weighted allele score and *P* = 0.22 for the simple allele score).

### Meta-analyses of individual genetic variants for dementia

**Fig. 3:**
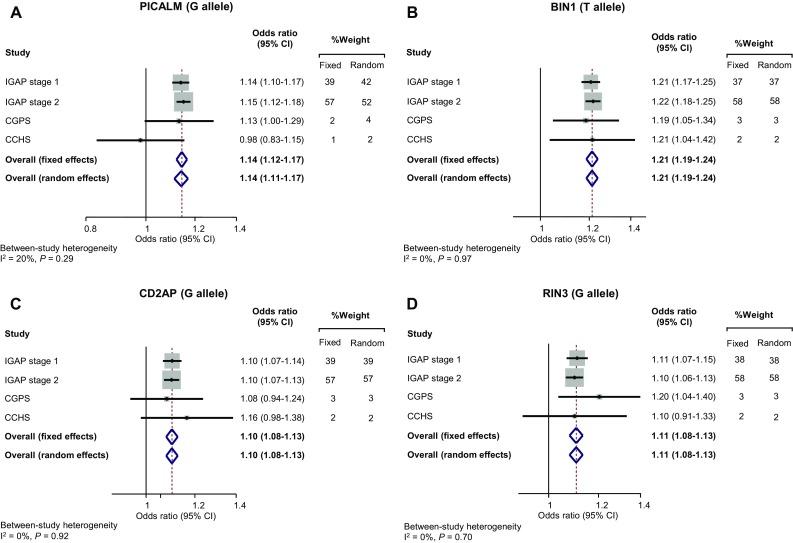
Meta-analysis of *PICALM*, *BIN1*, *CD2AP*, and *RIN3* in IGAP 1 and 2, CGPS, and CCHS. Summarizing risk of Alzheimer’s disease by risk increasing alleles in *PICALM* (G allele), *BIN1* (T allele), *CD2AP* (G allele), and *RIN3* (G allele). Horizontal lines correspond to 95% confidence intervals by forest plots. Diamonds and broken vertical lines represent summary estimates. Confidence intervals for the summary estimates correspond to the width of the diamonds. Grey shaded areas correspond to the weights of the studies in the meta-analysis from the fixed effects models (right column). *P* for heterogeneity from Cochran’s Q test. *APOE *= apolipoprotein E gene; *APOE* genotype = ε2/ε3/ε4 *APOE* genotype; *BIN1 *= bridging integrator 1; CCHS = the Copenhagen City Heart Study; *CD2AP *= CD2-associated protein; CGPS = the Copenhagen General Population Study; CI = confidence interval; IGAP = the International Genomics of Alzheimer’s Project; *PICALM *= phosphatidylinositol-binding clathrin assembly protein; *RIN3 *= Ras and Rab interactor 3

Meta-analyses included the present two prospective cohorts of the Danish general population and stage 1 and 2 from IGAP which includes meta-analyzed data from 15 individual studies [16]. For *PICALM* overall fixed- and random-effects odds ratios were 1.14 (1.12–1.17) and 1.14 (1.11–1.17) with *I*^2^ of 20% (*P* for heterogeneity = 0.29) (Fig. 3, upper left panel). For *BIN1* odds ratios were 1.21 (1.19–1.24) for both overall fixed- and random-effects, with *I*^2^ of 0% (*P* for heterogeneity = 0.97) (Fig. 3, upper right panel). For *CD2AP* odds ratios were 1.10 (1.08–1.13) for both overall fixed- and random-effects, with *I*^2^ of 0% (*P* for heterogeneity = 0.92) (Fig. 3, lower left panel). For *RIN3* odds ratios were 1.11 (1.08–1.13) for both overall fixed- and random-effects, with *I*^2^ of 0% (*P* for heterogeneity = 0.70) (Fig. 3, lower right panel).

### Weighted/simple allele scores and risk of suggested vascular dementia and stroke

**Fig. 4:**
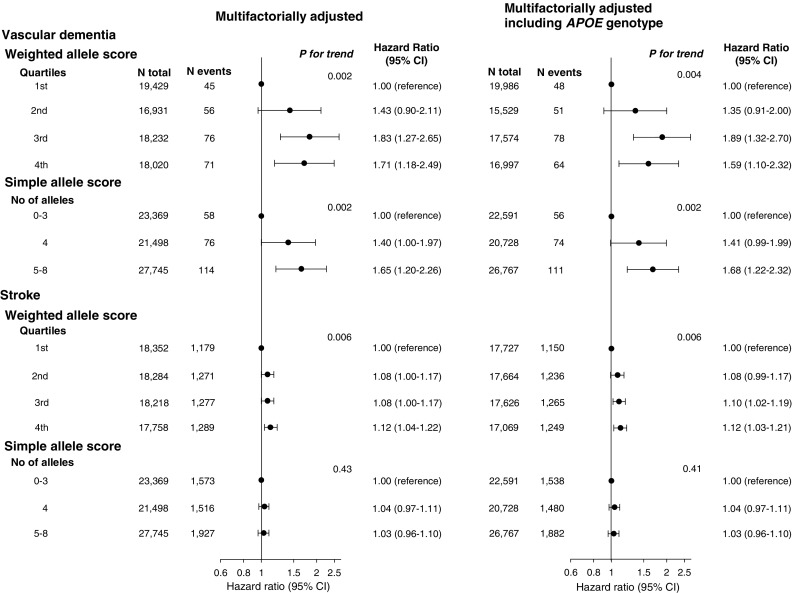
Risk of suggested vascular dementia and stroke as a function of weighted/simple allele scores. Individuals with suggested vascular dementia or stroke before blood sampling were excluded, leaving 72,612 individuals for analysis in the left panel. A total of 70,086 individuals with available *APOE* genotype were included in the right panel. Hazard ratios were multifactorially adjusted for age (as time scale), sex, hypertension, diabetes, smoking, alcohol intake, physical inactivity, postmenopausal status and hormonal replacement therapy in women, lipid-lowering therapy and educational level. Right panel additionally includes adjustment for *APOE* genotype. *P* for trend from Cox regression. *APOE *= apolipoprotein E gene; *APOE* genotype = ε2/ε3/ε4 *APOE* genotype; CI = confidence interval

Multifactorially adjusted hazard ratios for the fourth versus the first weighted allele score quartile were 1.71 (1.18–2.49) for suggested vascular dementia and 1.12 (1.04–1.22) for stroke. When prevalent stroke was excluded, risk of suggested vascular dementia remained (fourth versus first quartile 2.01 (1.32–3.07), data not shown). For the simple allele score multifactorially adjusted hazard ratios for 5–8 alleles versus 0–3 alleles were 1.65 (1.20–2.26) for suggested vascular dementia and 1.03 (0.96–1.10) for stroke (Fig. 4, left panel). Results were similar after further adjustment for *APOE* genotype (Fig. 4, right panel), and when excluding participants who developed dementia of any subtype within the first six months of follow-up (Supplementary Figure 10).

### Blood pressure, heart rate and biochemical characteristics of study participants by weighted allele score

Weighted allele score quartiles for all endpoints, were not associated with any variation in vital signs (systolic and diastolic blood pressure, heart rate), plasma levels of electrolytes (potassium, sodium, chloride), renal function (creatinine, estimated glomerular filtration rate), liver function (alanine aminotransferase, alkaline phosphatase, bilirubin, gamma-glutamyl transpeptidase), glucose level or high-sensitivity C-reactive protein (Supplementary Tables 4 and 5 for Alzheimer’s disease and all dementia; data not shown for suggested vascular dementia and stroke).

## Discussion

The principal findings of this study are that genetic variation in *PICALM*, *BIN1*, *CD2AP* and *RIN3*, four genes suggested to be involved in blood–brain barrier amyloid-β transcytosis, are associated with increased risk of Alzheimer’s disease, all dementia, and suggested vascular dementia in the general population independent of the strong *APOE* genotype. There was no conclusive association with risk of stroke. These findings may suggest that compromising blood–brain barrier function has implications for both brain tissue—manifested by increased Alzheimer’s disease risk—as well as for cerebral vasculature—manifested by increased risk of suggested vascular dementia.

To our knowledge this is the first study to simultaneously assess risk of dementia and stroke as a function of genetic variation in all four, potential blood–brain barrier transcytosis genes. *PICALM* has consistently been associated with Alzheimer’s disease in several GWA studies with risk estimates in the range of 1.14–1.27 [16, 30, 33, 34]. *BIN1* in the range of 1.15–1.36 [16, 31, 33–35, 37], *CD2AP* in the range of 1.10–1.12 [16, 31, 37], and finally, *RIN3* with an odds ratio for the risk increasing allele of 1.10 [16]. The present findings of these genes with suggested vascular dementia are however novel.

The biological mechanisms of our findings remain to be determined and the evidence from population studies linking the four selected variants to blood–brain barrier amyloid-β transcytosis pathways is limited. Recently new light was shed on the function of *PICALM* in Alzheimer’s disease pathology, emphasizing clathrin-mediated endocytosis as a potential important mechanism in amyloid-β clearance across the blood–brain barrier [29]. Zhao et al. showed that reductions of PICALM in brain endothelium in Alzheimer’s disease correlated with amyloid-β accumulation, Alzheimer’s disease neuropathology, and cognitive impairment in mice. *Picalm*^+*/*−^ mice had a reduction in amyloid-β40 and amyloid-β42 efflux across the blood–brain barrier of 41% and 61%, respectively, compared to littermate controls. Using in vitro endothelial monolayer to mimic the blood–brain barrier, they found that amyloid-β binding to LRP1 enhanced the binding of PICALM, which initiated PICALM/clathrin-dependent endocytosis of the amyloid-β-LRP1 complex and subsequent transcytosis involving GTPases Rab5 and Rab11 controlling, respectively, early endosome formation and vesicle exocytosis [55–60]. In primarily in vitro studies both BIN1 and CD2AP are shown to bind to Rab5 [27], and RIN3 is shown to mediate the recruitment of BIN1 and CD2AP to Rab5-positive early endosomes in the endocytic transport pathway [24, 27]. Taken together, these findings suggest that PICALM and interacting proteins control amyloid-β transport across the blood–brain barrier and clearance of amyloid-β from brain through clathrin-mediated endocytosis. Therefore, it may be biologically plausible that inactivating genetic variants in genes involved in this transcytosis pathway may cause an increased risk of Alzheimer’s disease due to accumulation of amyloid-β in brain tissue. This is also supported by a recent pathway analysis pinpointing endocytosis and clathrin/AP2-adaptor complex as important mechanisms in dementia [61–63].

Vascular dementia is a disorder in the blood supply of the brain, caused by preceding hemorrhage or ischemia located in either larger vessels or in the microvasculature [64]. Accumulation of amyloid-β in the vessel walls of the blood–brain barrier could lead to microinfarcts or microbleeds eventually causing vascular dementia but not necessarily clinically overt stroke. The present findings may emphasize the blood–brain barrier as a delicate border structure between brain and vasculature and supports the now widespread understanding of mixed pathology in most dementia cases. Thus, it is biologically meaningful that disruptions in this pathway affect both brain and vascular disease, in contrast to other dementia susceptibility genes, that exert their effects only in brain [65, 66].

An important limitation to this study is that endpoints are based on ICD registry codes from hospitals and death certificates diagnosed in routine clinical practice and thus only captures individuals in contact with hospitals. This is in contrast to research studies where all individuals living in one area are examined and diagnosed with use of standardized instruments, trained staff and standardized diagnostic methods [67]. Consequently, an inherent limitation of using registry-based diagnoses is underdiagnosis. Another limitations is that even though the national Danish registries are regarded among the best of its kind [43, 44], and the quality of the Danish registry-based dementia diagnoses previously has been validated, including a full clinical workup performed by dementia experts [48], the subtypes of dementia, especially vascular dementia and other rare forms, are uncertain [48]. Therefore, we have emphasized throughout that the diagnostic uncertainty should be considered when interpreting the results for vascular dementia. Consequently, we named this subtype “suggested vascular dementia” throughout the paper. Another potential limitation is the uncertainty of age at onset for dementia diseases. It is commonly accepted that for dementia prodromal phases can last for decades. Hence, we cannot exclude that some of the participants receiving a dementia diagnosis during our follow-up time already have dementia pathology at baseline.

Strengths of this study are the prospective design and the large, well-characterized, ethnically homogeneous cohort of the general population with no losses to follow-up. Furthermore, no differences of participant characteristics across weighted/simple allele score groups were observed and therefore obvious confounding of the gene scores could be excluded. Furthermore, there was no interaction between the genetic scores and age at baseline, age at diagnosis, or *APOE* genotype. Due to the extensive phenotyping of biochemical and other quantities in the present cohorts, we could also evaluate blood pressure, heart rate, electrolytes, and measures of kidney function, liver function, glucose metabolism and inflammation. We found no significant variation across weighted/simple allele score groups, suggesting that this molecular pathway may be safe to target therapeutically. The sufficient quality of the Danish registry-based all dementia and Alzheimer’s disease diagnoses was further supported by the well-known association with the apolipoprotein E ε4 allele in the present cohorts [42] and by the present meta-analyses for each individual SNP showing similar estimates in our cohorts as those found by the IGAP [16]. Diagnoses of stroke in our cohorts have been validated by trained physicians using the WHO definition of cerebrovascular disease, as previously described [68].

In conclusion, by combining common genetic variation in four genes with possible function in the blood–brain barrier, we found an increased risk of Alzheimer’s disease, all dementia and suggested vascular dementia with increasing weighted/simple allele score groups independent of the *APOE* ε4 allele. These findings may suggest that clathrin-mediated endocytosis in clearance of amyloid-β across the blood–brain barrier is important for the integrity of both brain tissue and cerebral vessels.

## Electronic supplementary material

Below is the link to the electronic supplementary material.

Supplementary material 1 (DOCX 631 kb)
