# Supplementary material for: Blood–brain barrier transcytosis genes, risk of dementia and stroke: a prospective cohort study of 74,754 individuals
Source: Eur J Epidemiol. 2019 Mar 4;34(6):579–90. doi: 10.1007/s10654-019-00498-2 (PMC6497814; doi:10.1007/s10654-019-00498-2)

**Supplementary Material**

**Blood-brain barrier transcytosis genes, risk of dementia and stroke: a prospective cohort study of 74,754 individuals**

Ida Juul Rasmussen, Anne Tybjærg-Hansen, Katrine Laura Rasmussen, Børge G. Nordestgaard, Ruth Frikke-Schmidt

**Supplementary Table 1:** Single per allele weights (part A) used for dementia increasing weighted allele score calculation (part B)

**Supplementary Table 2:** Characteristics of study participants by disease status

**Supplementary Table 3:** Age at diagnosis of Alzheimer’s disease, all dementia, suggested vascular dementia and stroke

**Supplementary Table 4:** Vital signs and biochemistry of study participants by weighted allele score quartile for Alzheimer’s disease

**Supplementary Table 5:** Vital signs and biochemistry of study participants by weighted allele score quartile for all dementia

**Supplementary Figure 1:** Distribution of age at baseline for the combined cohort, the CGPS and the CCHS

**Supplementary Figure 2:** Distribution of follow-up time for the combined cohort, the CGPS and the CCHS

**Supplementary Figure 3:** Risk of Alzheimer’s disease as a function of weighted allele score quartiles

**Supplementary Figure 4:** Risk of Alzheimer’s disease and all dementia by weighted/simple allele scores in the CGPS

**Supplementary Figure 5:** Risk of Alzheimer’s disease and all dementia by weighted/simple allele scores in the CCHS

**Supplementary Figure 6:** Risk of Alzheimer’s disease and all dementia by weighted/simple allele scores after exclusion of participants diagnosed with dementia within the first six months from baseline

**Supplementary Figure 7:** Risk of Alzheimer’s disease and all dementia by weighted allele score in tertiles

**Supplementary Figure 8:** Risk of Alzheimer’s disease and all dementia stratified on *APOE* ε4 non-carriers and *APOE* ε4 carriers

**Supplementary Figure 9:** Risk of Alzheimer’s disease and all dementia stratified on age groups

**Supplementary Figure 10:** Risk of suggested vascular dementia and stroke by weighted/simple allele scores after exclusion of participants diagnosed with dementia within the first six months from baseline

***Supplementary Table 1.* Single per allele weights (part A) used for dementia increasing weighted allele score calculation (part B)**

**A) Single per allele weights for Alzheimer’s disease**

| Gene | rs number | Minor allele | Common allele | Alzheimer’s disease risk increasing allele | Single per allele weights (CGPS+CCHS) | Single per  allele weights (GWAS)^1^ |
| --- | --- | --- | --- | --- | --- | --- |
| *PICALM* | rs10792832 | A | G | G | 0.04354788 | 0.13976194 |
| *BIN1* | rs6733839 | T | C | T | 0.15352334 | 0.19885086 |
| *CD2AP* | rs10948363 | G | A | G | 0.08516618 | 0.09531018 |
| *RIN3* | rs10498633 | T | G | G | 0.10495894 | 0.09531018 |

The single allele weights correspond to the per-Alzheimer’s disease risk increasing allele regression-coefficients adjusted for age and sex. *BIN1*=bridging integrator 1; *CD2AP*=CD2-associated protein; *PICALM*=phosphatidylinositol-binding clathrin assembly protein; *RIN3*=Ras and Rab interactor 3.

**A) Single per allele weights for all dementia**

| Gene | rs number | Minor allele | Common allele | Dementia risk increasing allele | Single per allele weights (CGPS+CCHS) | Single per allele weights (GWAS)^1^ |
| --- | --- | --- | --- | --- | --- | --- |
| *PICALM* | rs10792832 | A | G | G | 0.06599184 | NA |
| *BIN1* | rs6733839 | T | C | T | 0.12566919 | NA |
| *CD2AP* | rs10948363 | G | A | G | 0.03784669 | NA |
| *RIN3* | rs10498633 | T | G | G | 0.09335099 | NA |

The single allele weights correspond to the per-all dementia disease risk increasing allele regression-coefficients adjusted for age and sex. *BIN1*=bridging integrator 1; *CD2AP*=CD2-associated protein; NA= Not available; *PICALM*=phosphatidylinositol-binding clathrin assembly protein; *RIN3*=Ras and Rab interactor 3.

**B) Combined single allele weights into Alzheimer’s disease risk increasing weighted allele score**

| *PICALM* | *BIN1* | *CD2AP* | *RIN3* | Weighted allele score | Weighted allele score quartile | N |
| --- | --- | --- | --- | --- | --- | --- |
| AA | CC | AA | TT | 0 | 1 | 128 |
| GA | CC | AA | TT | 0.0435479 | 1 | 416 |
| AA | CC | GA | TT | 0.0851662 | 1 | 86 |
| GG | CC | AA | TT | 0.0870958 | 1 | 298 |
| AA | CC | AA | TG | 0.1049589 | 1 | 718 |
| GA | CC | GA | TT | 0.1287141 | 1 | 277 |
| GA | CC | AA | TG | 0.1485068 | 1 | 2,446 |
| AA | TC | AA | TT | 0.1535233 | 1 | 144 |
| AA | CC | GG | TT | 0.1703324 | 1 | 24 |
| GG | CC | GA | TT | 0.1722619 | 1 | 220 |
| AA | CC | GA | TG | 0.1901251 | 1 | 544 |
| GG | CC | AA | TG | 0.1920547 | 1 | 2,043 |
| GA | TC | AA | TT | 0.1970712 | 1 | 529 |
| AA | CC | AA | GG | 0.2099179 | 1 | 1,219 |
| GA | CC | GG | TT | 0.2138802 | 1 | 55 |
| GA | CC | GA | TG | 0.233673 | 1 | 1,800 |
| AA | TC | GA | TT | 0.2386895 | 1 | 100 |
| GG | TC | AA | TT | 0.2406191 | 1 | 429 |
| GA | CC | AA | GG | 0.2534658 | 1 | 4,202 |
| GG | CC | GG | TT | 0.2574281 | 1 | 39 |
| AA | TC | AA | TG | 0.2584823 | 1 | 984 |
| AA | CC | GG | TG | 0.2752913 | 1 | 98 |
| GG | CC | GA | TG | 0.2772209 | 1 | 1,437 |
| GA | TC | GA | TT | 0.2822374 | 1 | 369 |
| AA | CC | GA | GG | 0.2950841 | 1 | 818 |
| GG | CC | AA | GG | 0.2970136 | 2 | 3,364 |
| GA | TC | AA | TG | 0.3020301 | 2 | 3,309 |
| AA | TT | AA | TT | 0.3070467 | 2 | 50 |
| GA | CC | GG | TG | 0.3188392 | 2 | 329 |
| AA | TC | GG | TT | 0.3238557 | 2 | 19 |
| GG | TC | GA | TT | 0.3257853 | 2 | 282 |
| GA | CC | GA | GG | 0.3386319 | 2 | 2,883 |
| AA | TC | GA | TG | 0.3436485 | 2 | 681 |
| GG | TC | AA | TG | 0.345578 | 2 | 2,730 |
| GA | TT | AA | TT | 0.3505946 | 2 | 150 |
| GG | CC | GG | TG | 0.3623871 | 2 | 266 |
| AA | TC | AA | GG | 0.3634412 | 2 | 1,585 |
| GA | TC | GG | TT | 0.3674036 | 2 | 70 |
| AA | CC | GG | GG | 0.3802502 | 2 | 145 |
| GG | CC | GA | GG | 0.3821798 | 2 | 2,388 |
| GA | TC | GA | TG | 0.3871963 | 3 | 2,389 |
| AA | TT | GA | TT | 0.3922129 | 3 | 38 |
| GG | TT | AA | TT | 0.3941424 | 3 | 143 |
| GA | TC | AA | GG | 0.4069891 | 3 | 5,178 |
| GG | TC | GG | TT | 0.4109515 | 3 | 45 |
| AA | TT | AA | TG | 0.4120056 | 3 | 309 |
| GA | CC | GG | GG | 0.4237981 | 3 | 533 |
| AA | TC | GG | TG | 0.4288146 | 3 | 123 |
| GG | TC | GA | TG | 0.4307442 | 3 | 2,000 |
| GA | TT | GA | TT | 0.4357607 | 3 | 110 |
| AA | TC | GA | GG | 0.4486074 | 3 | 1,113 |
| GG | TC | AA | GG | 0.450537 | 3 | 4,487 |
| GA | TT | AA | TG | 0.4555535 | 3 | 1,103 |
| GG | CC | GG | GG | 0.467346 | 3 | 420 |
| GA | TC | GG | TG | 0.4723625 | 3 | 418 |
| AA | TT | GG | TT | 0.4773791 | 4 | 4 |
| GG | TT | GA | TT | 0.4793086 | 4 | 104 |
| GA | TC | GA | GG | 0.4921553 | 4 | 3,755 |
| AA | TT | GA | TG | 0.4971718 | 4 | 222 |
| GG | TT | AA | TG | 0.4991014 | 4 | 868 |
| GG | TC | GG | TG | 0.5159104 | 4 | 337 |
| AA | TT | AA | GG | 0.5169646 | 4 | 460 |
| GA | TT | GG | TT | 0.5209269 | 4 | 34 |
| AA | TC | GG | GG | 0.5337736 | 4 | 208 |
| GG | TC | GA | GG | 0.5357032 | 4 | 3,270 |
| GA | TT | GA | TG | 0.5407197 | 4 | 798 |
| GA | TT | AA | GG | 0.5605124 | 4 | 1,754 |
| GG | TT | GG | TT | 0.5644748 | 4 | 25 |
| GA | TC | GG | GG | 0.5773215 | 4 | 651 |
| AA | TT | GG | TG | 0.582338 | 4 | 44 |
| GG | TT | GA | TG | 0.5842676 | 4 | 685 |
| AA | TT | GA | GG | 0.6021307 | 4 | 365 |
| GG | TT | AA | GG | 0.6040603 | 4 | 1,447 |
| GG | TC | GG | GG | 0.6208693 | 4 | 605 |
| GA | TT | GG | TG | 0.6258858 | 4 | 150 |
| GA | TT | GA | GG | 0.6456786 | 4 | 1,277 |
| GG | TT | GG | TG | 0.6694337 | 4 | 135 |
| AA | TT | GG | GG | 0.6872969 | 4 | 63 |
| GG | TT | GA | GG | 0.6892265 | 4 | 974 |
| GA | TT | GG | GG | 0.7308448 | 4 | 227 |
| GG | TT | GG | GG | 0.7743927 | 4 | 209 |

For all existing genotype combinations their single allele weights were summarized into a weighted allele score. These weighted allele scores were subsequently categorized into quartiles of approximate equal size and numbered 1 to 4 with increasing risk of Alzheimer’s disease. Risk increasing alleles are marked in red. *BIN1*=bridging integrator 1; *CD2AP*=CD2-associated protein; *PICALM*=phosphatidylinositol-binding clathrin assembly protein; *RIN3*=Ras and Rab interactor 3.

**B) Combined single allele weights into all dementia risk increasing weighted allele score**

| *PICALM* | *BIN1* | *CD2AP* | *RIN3* | Weighted allele score | Weighted allele score quartile | N |
| --- | --- | --- | --- | --- | --- | --- |
| AA | CC | AA | TT | 0 | 1 | 128 |
| AA | CC | GA | TT | 0.0378467 | 1 | 86 |
| GA | CC | AA | TT | 0.0659918 | 1 | 416 |
| AA | CC | GG | TT | 0.0756934 | 1 | 24 |
| AA | CC | AA | TG | 0.093351 | 1 | 718 |
| GA | CC | GA | TT | 0.1038385 | 1 | 277 |
| AA | TC | AA | TT | 0.1256692 | 1 | 144 |
| AA | CC | GA | TG | 0.1311977 | 1 | 544 |
| GG | CC | AA | TT | 0.1319837 | 1 | 298 |
| GA | CC | GG | TT | 0.1416852 | 1 | 55 |
| GA | CC | AA | TG | 0.1593428 | 1 | 2,446 |
| AA | TC | GA | TT | 0.1635159 | 1 | 100 |
| AA | CC | GG | TG | 0.1690444 | 1 | 98 |
| GG | CC | GA | TT | 0.1698304 | 1 | 220 |
| AA | CC | AA | GG | 0.186702 | 1 | 1,219 |
| GA | TC | AA | TT | 0.191661 | 1 | 529 |
| GA | CC | GA | TG | 0.1971895 | 1 | 1,800 |
| AA | TC | GG | TT | 0.2013626 | 1 | 19 |
| GG | CC | GG | TT | 0.2076771 | 1 | 39 |
| AA | TC | AA | TG | 0.2190202 | 1 | 984 |
| AA | CC | GA | GG | 0.2245487 | 1 | 818 |
| GG | CC | AA | TG | 0.2253347 | 1 | 2,043 |
| GA | TC | GA | TT | 0.2295077 | 1 | 369 |
| GA | CC | GG | TG | 0.2350362 | 1 | 329 |
| AA | TT | AA | TT | 0.2513384 | 1 | 50 |
| GA | CC | AA | GG | 0.2526938 | 1 | 4,202 |
| AA | TC | GA | TG | 0.2568669 | 1 | 681 |
| GG | TC | AA | TT | 0.2576529 | 1 | 429 |
| AA | CC | GG | GG | 0.2623954 | 2 | 145 |
| GG | CC | GA | TG | 0.2631814 | 2 | 1,437 |
| GA | TC | GG | TT | 0.2673544 | 2 | 70 |
| GA | TC | AA | TG | 0.285012 | 2 | 3,309 |
| AA | TT | GA | TT | 0.2891851 | 2 | 38 |
| GA | CC | GA | GG | 0.2905405 | 2 | 2,883 |
| AA | TC | GG | TG | 0.2947136 | 2 | 123 |
| GG | TC | GA | TT | 0.2954996 | 2 | 282 |
| GG | CC | GG | TG | 0.301028 | 2 | 266 |
| AA | TC | AA | GG | 0.3123712 | 2 | 1,585 |
| GA | TT | AA | TT | 0.3173302 | 2 | 150 |
| GG | CC | AA | GG | 0.3186857 | 2 | 3,364 |
| GA | TC | GA | TG | 0.3228587 | 2 | 2,389 |
| AA | TT | GG | TT | 0.3270318 | 2 | 4 |
| GA | CC | GG | GG | 0.3283872 | 2 | 533 |
| GG | TC | GG | TT | 0.3333462 | 2 | 45 |
| AA | TT | AA | TG | 0.3446894 | 2 | 309 |
| AA | TC | GA | GG | 0.3502178 | 2 | 1,113 |
| GG | TC | AA | TG | 0.3510039 | 2 | 2,730 |
| GA | TT | GA | TT | 0.3551769 | 3 | 110 |
| GG | CC | GA | GG | 0.3565323 | 3 | 2,388 |
| GA | TC | GG | TG | 0.3607054 | 3 | 418 |
| GA | TC | AA | GG | 0.378363 | 3 | 5,178 |
| AA | TT | GA | TG | 0.3825361 | 3 | 222 |
| GG | TT | AA | TT | 0.3833221 | 3 | 143 |
| AA | TC | GG | GG | 0.3880646 | 3 | 208 |
| GG | TC | GA | TG | 0.3888505 | 3 | 2,000 |
| GA | TT | GG | TT | 0.3930236 | 3 | 34 |
| GG | CC | GG | GG | 0.394379 | 3 | 420 |
| GA | TT | AA | TG | 0.4106812 | 3 | 1,103 |
| GA | TC | GA | GG | 0.4162097 | 3 | 3,755 |
| AA | TT | GG | TG | 0.4203827 | 3 | 44 |
| GG | TT | GA | TT | 0.4211687 | 3 | 104 |
| GG | TC | GG | TG | 0.4266973 | 3 | 337 |
| AA | TT | AA | GG | 0.4380403 | 4 | 460 |
| GG | TC | AA | GG | 0.4443549 | 4 | 4,487 |
| GA | TT | GA | TG | 0.4485279 | 4 | 798 |
| GA | TC | GG | GG | 0.4540564 | 4 | 651 |
| GG | TT | GG | TT | 0.4590154 | 4 | 25 |
| AA | TT | GA | GG | 0.4758871 | 4 | 365 |
| GG | TT | AA | TG | 0.476673 | 4 | 868 |
| GG | TC | GA | GG | 0.4822015 | 4 | 3,270 |
| GA | TT | GG | TG | 0.4863746 | 4 | 150 |
| GA | TT | AA | GG | 0.5040322 | 4 | 1,754 |
| AA | TT | GG | GG | 0.5137337 | 4 | 63 |
| GG | TT | GA | TG | 0.5145198 | 4 | 685 |
| GG | TC | GG | GG | 0.5200482 | 4 | 605 |
| GA | TT | GA | GG | 0.5418789 | 4 | 1,277 |
| GG | TT | GG | TG | 0.5523664 | 4 | 135 |
| GG | TT | AA | GG | 0.570024 | 4 | 1,447 |
| GA | TT | GG | GG | 0.5797256 | 4 | 227 |
| GG | TT | GA | GG | 0.6078708 | 4 | 974 |
| GG | TT | GG | GG | 0.6457174 | 4 | 209 |

For all existing genotype combinations their single allele weights were summarized into a weighted allele score. These weighted allele scores were subsequently categorized into quartiles of approximate equal size and numbered 1 to 4 with increasing risk of all dementia. Risk increasing alleles are marked in red. *BIN1*=bridging integrator 1; *CD2AP*=CD2-associated protein; *PICALM*=phosphatidylinositol-binding clathrin assembly protein; *RIN3*=Ras and Rab interactor 3.

***Supplementary Table 2.* Characteristics of study participants by disease status**

|  | **Without Alzheimer’s disease** | **Alzheimer’s**  **disease** | **Without all dementia** | **All dementia** |
| --- | --- | --- | --- | --- |
| No. of individuals (%) | 73,414 (98.2) | 1,340 (1.8) | 72,240 (96.6) | 2,514 (3.7) |
| Age (years) | 57 (47-67) | 74 (69-79)* | 57 (47-66) | 75 (69-80)* |
| Female (%) | 55 | 61* | 55 | 58* |
| Total cholesterol (mmol/L) | 5.6 (4.9-6.4) | 5.9 (5.2-6.7)* | 5.6 (4.9-6.4) | 5.9 (5.2-6.7)* |
| LDL cholesterol (mmol/L) | 3.2 (2.6-3.9) | 3.4 (2.8-4.2)* | 3.2 (2.6-3.9) | 3.5 (2.7-4.2)* |
| HDL cholesterol (mmol/L) | 1.5 (1.2-1.9) | 1.7 (1.4-2.0)* | 1.5 (1.2-1.9) | 1.6 (1.3-2.0)* |
| Triglycerides (mmol/L) | 1.4 (1.0-2.1) | 1.5 (1.1-2.1)* | 1.4 (1.0-2.1) | 1.5 (1.1-2.1)* |
| Body mass index (kg/m^2^) | 26 (23-28) | 26 (23-28) | 26 (23-28) | 26 (23-28) |
| Hypertension (%) | 58 | 77* | 57 | 77* |
| Diabetes mellitus (%) | 4 | 6* | 4 | 7* |
| Smoking (%) | 24 | 21† | 24 | 26† |
| High alcohol consumption (%) | 18 | 16† | 18 | 16 |
| Physical inactivity (%) | 52 | 56† | 52 | 62* |
| Postmenopausal (%)‡ | 66 | 98* | 65 | 99* |
| Hormonal replacement therapy (%)‡ | 11 | 16* | 11 | 15* |
| Lipid-lowering therapy (%) | 9 | 16* | 9 | 14* |
| Education < 8 years (%) | 14 | 37* | 13 | 38* |

Values are median (interquartile range) or percent and are from the day of enrolment (2003 and onwards for the CGPS and 1991-1994 or 2001-2003 for the CCHS). Hypertension was use of anti-hypertensive medication, a systolic blood pressure of 140 mm Hg or greater, and/or a diastolic blood pressure of 90 mm Hg or greater. Diabetes mellitus was self-reported disease, use of insulin or oral hypoglycaemic agents, and/or non-fasting plasma glucose levels of more than 11 mmol/L (>198 mg/dL). Smoking was current smoking. High alcohol consumption was >14/21 units per week for women/men (1 unit=12 g alcohol, equivalent to one glass of wine or one beer (33 cL)). Physical inactivity was ≤four hours per week of light physical activity in leisure time. Women reported menopausal status and use of hormonal replacement therapy. Lipid-lowering therapy was primarily statins (yes/no), and low education was <8 years of education. *p<0.001 and †p<0.05 by Kruskal-Wallis one-way analysis of variance or Pearson’s χ^2^-test for Alzheimer’s disease versus without Alzheimer’s disease and for all dementia versus without all dementia. ‡In women only. HDL=high-density lipoprotein cholesterol; LDL=low-density lipoprotein cholesterol.

***Supplementary Table 3.* Age at diagnosis of Alzheimer’s disease, all dementia, suggested vascular dementia and stroke**

|  | **Alzheimer’s disease**  (N=1,340) | ***P* for trend** | **All dementia**  (N=2,514) | ***P* for trend** | **Suggested vascular dementia**  (N=248) | ***P* for trend** | **Stroke**  (N=5,016) | ***P* for trend** |
| --- | --- | --- | --- | --- | --- | --- | --- | --- |
| **Weighted allele score** |  | 0.93 |  | 0.45 |  | 0.71 |  | 0.90 |
| 1^st^ quartile (years) | 82 (76-86) |  | 83 (77-87) |  | 83 (76-88) |  | 75 (67-82) |  |
| 2^nd^ quartile (years) | 81 (77-86) |  | 82 (78-87) |  | 83 (76-89) |  | 75 (66-82) |  |
| 3^rd^ quartile (years) | 82 (76-86) |  | 82 (76-87) |  | 82 (78-88) |  | 76 (67-82) |  |
| 4^th^ quartile (years) | 81 (77-86) |  | 82 (77-87) |  | 82 (76-87) |  | 75 (67-82) |  |
| **Simple allele score**  0-3 alleles (years) | 82 (77-86) | 0.45 | 83 (77-87) | 0.20 | 83 (75-89) | 0.54 | 75 (67-83) | 0.79 |
| 4 alleles (years) | 82 (77-86) |  | 82 (77-87) |  | 84 (78-88) |  | 75 (66-82) |  |
| 5-8 alleles (years) | 81 (76-85) |  | 82 (76-87) |  | 82 (76-87) |  | 75 (67-82) |  |
| ***APOE* genotype** |  | 1*10^-4^ |  | 1*10^-4^ |  | 0.80 |  | 0.002 |
| ε33 | 83 (79-87) |  | 83 (78-88) |  | 83 (77-89) |  | 76 (67-83) |  |
| ε43 | 81 (75-85) |  | 81 (76-86) |  | 84 (76-88) |  | 74 (66-82) |  |
| ε32 | 84 (78-87) |  | 84 (79-89) |  | 84 (75-88) |  | 76 (67-83) |  |
| ε42 | 81 (76-84) |  | 82 (76-87) |  | 83 (77-84) |  | 73 (64-80) |  |
| ε44 | 78 (73-82) |  | 78 (73-83) |  | 80 (78-86) |  | 74 (67-80) |  |
| ε22 | 79 (72-81) |  | 77 (72-80) |  | - |  | 77 (67-86) |  |

For each column only, individuals diagnosed with the specific disease after baseline were included. Ages are age at diagnosis as registered in the national Danish Patient Registry and the national Danish Causes of Death Registry and are here listed as the median (interquartile range). There were no individuals with *APOE* genotype ε22 and suggested vascular dementia. Differences across weighted/simple allele score groups and *APOE* genotype were tested by Kruskal-Wallis one-way analysis of variance. *APOE*=Apolipoprotein E gene; *APOE* genotype=ε2/ε3/ε4 *APOE* genotype.

***Supplementary Table 4.* Vital signs and biochemistry of study participants by weighted allele score quartile for Alzheimer’s disease**

|  | **1st quartile** | **2nd quartile** | **3rd quartile** | **4th quartile** |
| --- | --- | --- | --- | --- |
| No. of individuals (%) | 19,423 (26) | 18,251 (24) | 18,409 (25) | 18,671 (25) |
| Systolic blood pressure (mmHg) | 139 (125-153) | 139 (125-154) | 139 (125-154) | 139 (125-153) |
| Diastolic blood pressure (mmHg) | 83 (76-90) | 83 (76-90) | 83 (76-90) | 83 (76-90) |
| Heart rate (beats/min) | 72 (65-80) | 73 (65-80) | 72 (65-80) | 73 (65-80) |
| Glucose (mmol/L) | 5.2 (4.7-5.7) | 5.1 (4.7-5.7) | 5.2 (4.7-5.7) | 5.1 (4.7-5.7) |
| Potassium (mmol/L) | 4.1 (3.9-4.3) | 4.1 (3.9-4.3) | 4.1 (3.9-4.3) | 4.1 (3.9-4.3) |
| Sodium (mmol/L) | 140 (139-142) | 140 (139-142) | 140 (139-142) | 140 (139-142) |
| Chloride (mmol/L) | 101 (99-103) | 101 (99-103) | 101 (99-103) | 101 (99-103) |
| Creatinine (μmol/L) | 83 (75-92) | 83 (75-92) | 83 (75-92) | 83 (75-92) |
| Estimated GFR (ml/min/1.73m^2^) | 78 (67-89) | 77 (67-89) | 78 (67-89) | 77 (67-89) |
| ALAT (U/L) | 20 (15-27) | 20 (15-27) | 20 (15-27) | 19 (15-27) |
| ALP (U/L) | 81 (68-97) | 81 (68-98) | 81 (68-98) | 82 (68-98) |
| Bilirubin (μmol/L) | 11 (8-14) | 11 (8-14) | 11 (8-14) | 11 (8-14) |
| GGT (U/L) | 28 (21-42) | 28 (21-42) | 28 (21-42) | 28 (21-42) |
| hsCRP (mg/L) | 1.5 (1.1-2.5) | 1.5 (1.1-2.5) | 1.5 (1.1-2.5) | 1.5 (1.1-2.6) |

Values are median (interquartile range) or percent and are from the day of enrolment (2003 and onwards for the CGPS and 1991-1994 or 2001-2003 for the CCHS). Information on heart rate was available in the CGPS. Estimated glomerular filtration rate was calculated according to CKD-EPIcrea^2^. Differences across weighted allele score groups were tested by Kruskal-Wallis one-way analysis of variance. ALAT=Alanine aminotransferase; ALP=Alkaline phosphatase; GFR=glomerular filtration rate; GGT=Gamma-glutamyl transpeptidase; hsCRP=high-sensitivity C-reactive protein.

***Supplementary Table 5.* Vital signs and biochemistry of study participants by weighted allele score quartile for all dementia**

|  | **1st quartile** | **2nd quartile** | **3rd quartile** | **4th quartile** |
| --- | --- | --- | --- | --- |
| No. of individuals (%) | 19,065 (26) | 19,772 (26) | 17,467 (23) | 18,450 (25) |
| Systolic BP (mmHg) | 139 (125-153) | 139 (125-154) | 139 (125-153) | 139 (125-153) |
| Diastolic BP (mmHg) | 83 (76-90) | 83 (76-90) | 83 (76-90) | 83 (76-90) |
| Heart rate (beats/min) | 72 (65-80) | 72 (65-80) | 72 (65-80) | 72 (65-80) |
| Glucose (mmol/L) | 5.2 (4.7-5.7) | 5.2 (4.7-5.7) | 5.1 (4.7-5.7) | 5.2 (4.7-5.7) |
| Potassium (mmol/L) | 4.1 (3.9-4.3) | 4.1 (3.9-4.3) | 4.1 (3.9-4.3) | 4.1 (3.9-4.3) |
| Sodium (mmol/L) | 140 (139-142) | 140 (139-142) | 140 (139-142) | 140 (139-142) |
| Chloride (mmol/L) | 101 (99-103) | 101 (99-103) | 101 (99-103) | 101 (99-103) |
| Creatinine (µmol/L) | 83 (75-92) | 83 (75-92) | 83 (75-92) | 83 (75-92) |
| Estimated GFR (ml/min/1.73m^2^) | 78 (67-89) | 77 (67-89) | 78 (67-89) | 77 (67-89) |
| ALAT (U/L) | 20 (15-27) | 20 (15-27) | 20 (15-27) | 19 (15-27) |
| ALP (U/L) | 81 (68-98) | 81 (68-97) | 81 (68-98) | 82 (68-98) |
| Bilirubin (µmol/L) | 11 (8-14) | 11 (8-14) | 11 (8-14) | 11 (8-14) |
| GGT (U/L) | 28 (21-42) | 28 (21-42) | 28 (21-42) | 28 (21-42) |
| hsCRP (mg/L) | 1.5 (1.1-2.5) | 1.5 (1.1-2.5) | 1.5 (1.1-2.5) | 1.5 (1.1-2.5) |

Values are median (interquartile range) or percent and are from the day of enrolment (2003 and onwards for the CGPS and 1991-1994 or 2001-2003 for the CCHS). Information on heart rate was available in the CGPS. Estimated glomerular filtration rate was calculated according to CKD-EPIcrea^2^. Differences across weighted allele score groups were tested by Kruskal-Wallis one-way analysis of variance. ALAT=Alanine aminotransferase; ALP=Alkaline phosphatase; GFR=glomerular filtration rate; GGT=Gamma-glutamyl transpeptidase; hsCRP=high-sensitivity C-reactive protein.

**Supplementary Figure legends**

***Supplementary Figure 1.* Distribution of age at baseline for the combined cohort, the CGPS and the CCHS**

Individuals with Alzheimer’s disease or all dementia before blood sampling were excluded, leaving 74,754 individuals for the age distribution for the combined cohort (top panel), 64,974 individuals for the age distribution for the CGPS (middle panel) and 9,780 individuals for the age distribution for the CCHS (bottom panel).

***Supplementary Figure 2.* Distribution of follow-up time for the combined cohort, the CGPS and the CCHS**

Individuals with Alzheimer’s disease or all dementia before blood sampling were excluded, leaving 74,754 individuals for the distribution of follow-up time for the combined cohort (top panel), 64,974 individuals for the distribution of follow-up time for the CGPS (middle panel) and 9,780 individuals for the distribution of follow-up time for the CCHS (bottom panel).

***Supplementary Figure 3.* Risk of Alzheimer’s disease as a function of weighted allele score quartiles**
Comparison of internally-derived weights and externally-derived weights^1^. Individuals with Alzheimer’s disease or all dementia before blood sampling were excluded, leaving 74,754 individuals for the analysis in the left and middle panel. A total of 72,138 with available *APOE* genotype were included in the right panel. Hazard ratios were adjusted for age (as time scale), and sex (left panel). In the middle panel hazard ratios were multifactorially adjusted for age (as time scale), sex, hypertension, diabetes, smoking, alcohol intake, physical inactivity, postmenopausal status and hormonal replacement therapy in women, lipid-lowering therapy and educational level. Right panel additionally includes adjustment for *APOE* genotype. Externally derived weights were derived from Lambert *et al.* *P* for trend from competing risks regression trend test. *APOE*=Apolipoprotein E gene; *APOE* genotype=ε2/ε3/ε4 *APOE* genotype; CI=confidence interval; GWAS=genome-wide association study.

***Supplementary Figure 4.* Risk of Alzheimer’s disease and all dementia by weighted/simple allele scores in the CGPS**
Individuals with Alzheimer’s disease or all dementia before blood sampling were excluded, leaving 64,974 individuals for the analysis in the left panel. A total of 62,383 with available *APOE* genotype were included in the right panel. Hazard ratios were multifactorially adjusted for age (as time scale), sex, hypertension, diabetes, smoking, alcohol intake, physical inactivity, postmenopausal status and hormonal replacement therapy in women, lipid-lowering therapy and educational level (left panel). Right panel additionally includes adjustment for *APOE* genotype. *P* for trend from competing risks regression trend test. *APOE*=Apolipoprotein E gene; *APOE* genotype=ε2/ε3/ε4 *APOE* genotype; CI=confidence interval; CGPS=the Copenhagen General Population Study.

***Supplementary Figure 5.* Risk of Alzheimer’s disease and all dementia by weighted/simple allele scores in the CCHS**
Individuals with Alzheimer’s disease or all dementia before blood sampling were excluded, leaving 9,780 individuals for the analysis in the left panel. A total of 9,755 with available *APOE* genotype were included in the right panel. Hazard ratios were multifactorially adjusted for age (as time scale), sex, hypertension, diabetes, smoking, alcohol intake, physical inactivity, postmenopausal status and hormonal replacement therapy in women, lipid-lowering therapy and educational level (left panel). Right panel additionally includes adjustment for *APOE* genotype. *P* for trend from competing risks regression trend test. *APOE*=Apolipoprotein E gene; *APOE* genotype=ε2/ε3/ε4 *APOE* genotype; CI=confidence interval; CCHS=the Copenhagen City Heart Study.

***Supplementary Figure 6.* Risk of Alzheimer’s disease and all dementia by weighted/simple allele scores after exclusion of participants diagnosed with dementia within the first six months from baseline**

Individuals with Alzheimer’s disease or all dementia before blood sampling or within the first six months from baseline were excluded, leaving 74,737 individuals for the analysis in the left panel. A total of 72,121 individuals with available *APOE* genotype were included in the middle panel. The right panel exclusively contains individuals with the *APOE* ε33 genotype (N=40,228). Hazard ratios were multifactorially adjusted for age (as time scale), sex, hypertension, diabetes, smoking, alcohol intake, physical inactivity, postmenopausal status and hormonal replacement therapy in women, lipid-lowering therapy and educational level. *P* for trend from competing risks regression trend test. *APOE*=Apolipoprotein E gene; *APOE* genotype=ε2/ε3/ε4 *APOE* genotype; CI=confidence interval.

***Supplementary Figure 7.* Risk of Alzheimer’s disease and all dementia by weighted allele score in tertiles**

Individuals with Alzheimer’s disease or all dementia before blood sampling were excluded, leaving 74,754 individuals for the analysis. Hazard ratios were multifactorially adjusted for age (as time scale), sex, hypertension, diabetes, smoking, alcohol intake, physical inactivity, postmenopausal status and hormonal replacement therapy in women, lipid-lowering therapy and educational level. *P* for trend from competing risks regression trend test. *APOE*=Apolipoprotein E gene; *APOE* genotype=ε2/ε3/ε4 *APOE* genotype; CI=confidence interval.

***Supplementary Figure 8.* Risk of Alzheimer’s disease and all dementia stratified on *APOE* ε4 non-carriers and *APOE* ε4 carriers**

Individuals with no available *APOE* genotype or Alzheimer’s disease or all dementia before blood sampling were excluded. Analysis in the left panel only included individuals carrying no *APOE* ε4 alleles (N=49,696). For the analysis in the right panel only individuals carrying at least one *APOE* ε4 allele were included (N=22,442). Hazard ratios were multifactorially adjusted for age (as time scale), sex, hypertension, diabetes, smoking, alcohol intake, physical inactivity, postmenopausal status and hormonal replacement therapy in women, lipid-lowering therapy and educational level. *P* for trend from competing risks regression trend test. *APOE*=Apolipoprotein E gene; *APOE* genotype=ε2/ε3/ε4 *APOE* genotype; CI=confidence interval.

***Supplementary Figure 9.* Risk of Alzheimer’s disease and all dementia stratified on age groups**

Individuals with Alzheimer’s disease or all dementia before blood sampling were excluded. Analysis in the left panel only included individuals younger than 65 years of age (N=52,083). In the middle panel analysis only included individuals aged 65-80 years (N=19,185) and analysis in the left panel only included individuals aged 80 years or older (N=3,486). Hazard ratios were multifactorially adjusted for age (as time scale), sex, hypertension, diabetes, smoking, alcohol intake, physical inactivity, postmenopausal status and hormonal replacement therapy in women, lipid-lowering therapy and educational level. *P* for trend from competing risks regression trend test. *APOE*=Apolipoprotein E gene; *APOE* genotype=ε2/ε3/ε4 *APOE* genotype; CI=confidence interval.

***Supplementary Figure 10.* Risk of suggested vascular dementia and stroke by weighted/simple allele scores after exclusion of participants diagnosed with dementia within the first six months from baseline**

Individuals with all dementia or stroke before blood sampling were excluded. Individuals with all dementia within the first six months from baseline were also excluded, leaving 72,516 individuals for the analysis in the left panel. A total of 69,995 individuals with available *APOE* genotype were included in the right panel. Hazard ratios were multifactorially adjusted for age (as time scale), sex, hypertension, diabetes, smoking, alcohol intake, physical inactivity, postmenopausal status and hormonal replacement therapy in women, lipid-lowering therapy and educational level. *P* for trend from competing risks regression trend test. *APOE*=Apolipoprotein E gene; *APOE* genotype=ε2/ε3/ε4 *APOE* genotype; CI=confidence interval.

**Supplementary references**

1. Lambert JC, Ibrahim-Verbaas CA, Harold D, et al. Meta-analysis of 74,046 individuals identifies 11 new susceptibility loci for Alzheimer’s disease. *Nat Genet*. 2013;45(12):1452-1458. doi:10.1038/ng.2802.

2. Kidney Disease: Improving Global Outcomes (KDIGO) CKD Group. KDIGO 2012 Clinical Practice Guideline for the Evaluation and Management of Cronic Kidney Disease. *Kidney Int Suppl*. 2013;3:1-150.

**Supplementary Figure 1. Distribution of age at baseline for the combined cohort, the CGPS and the CCHS**


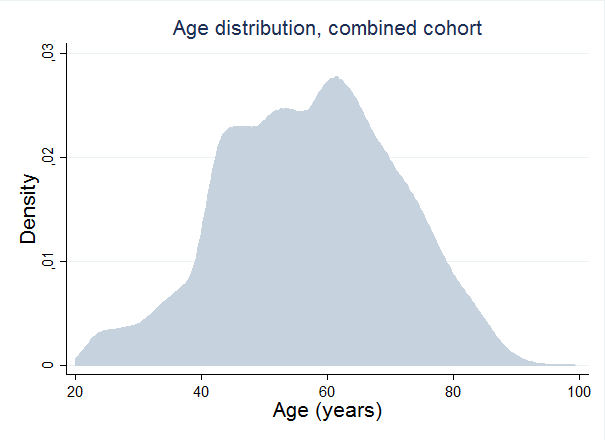

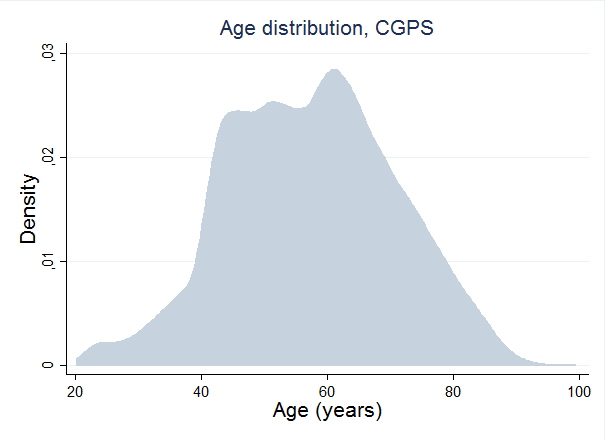

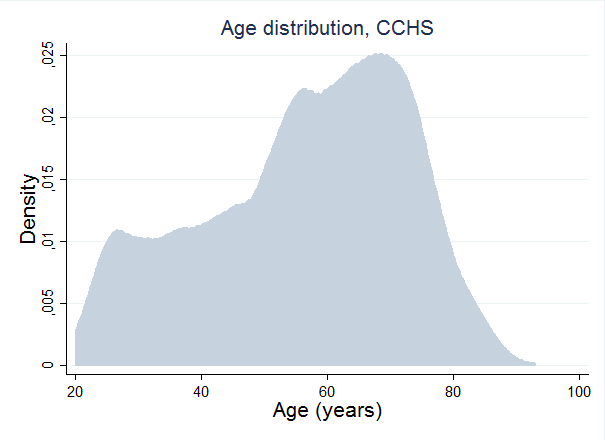


**Supplementary Figure 2. Distribution of follow-up time for the combined cohort, the CGPS and the CCHS**

**
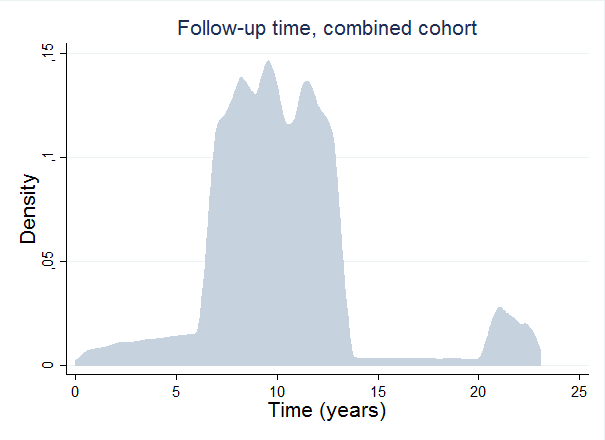

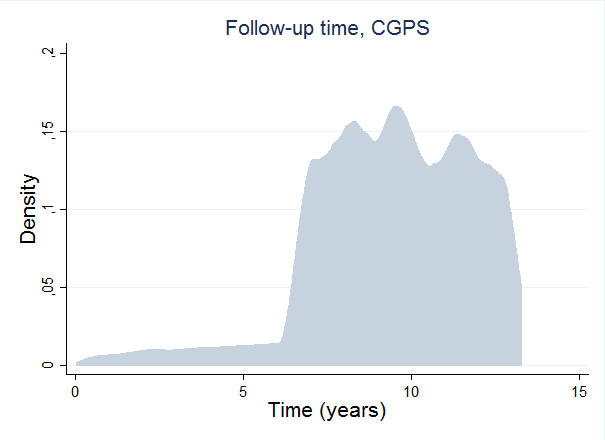

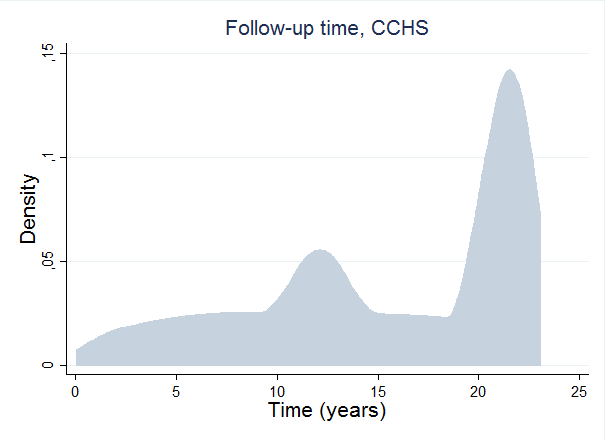
**

Entering at the 1991-94 examination

Entering at the 2001-03 examination


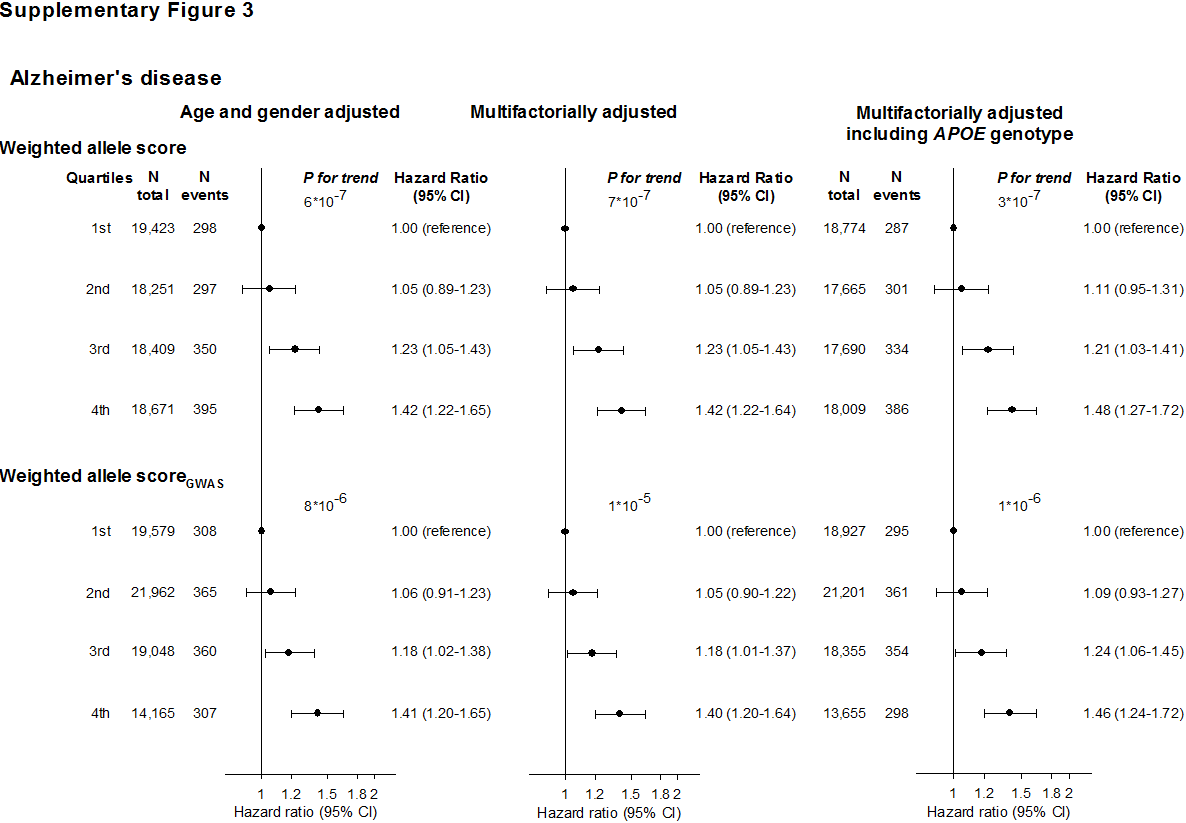


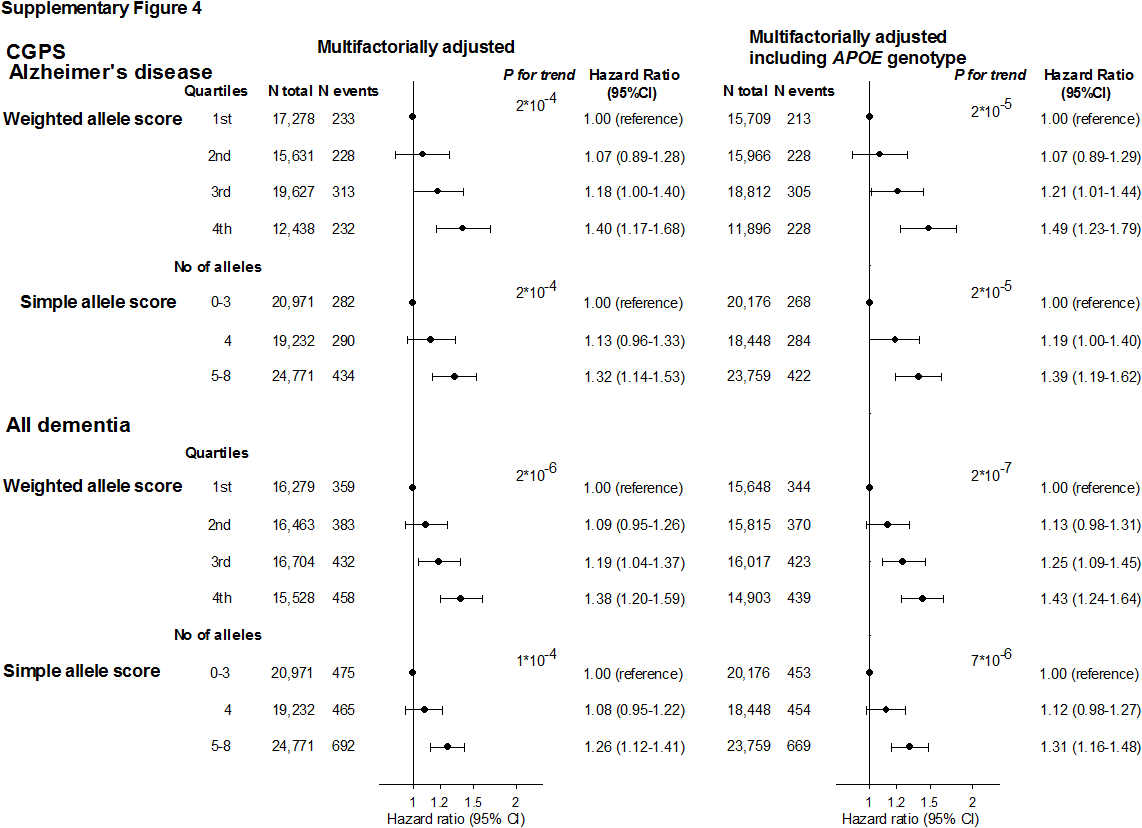


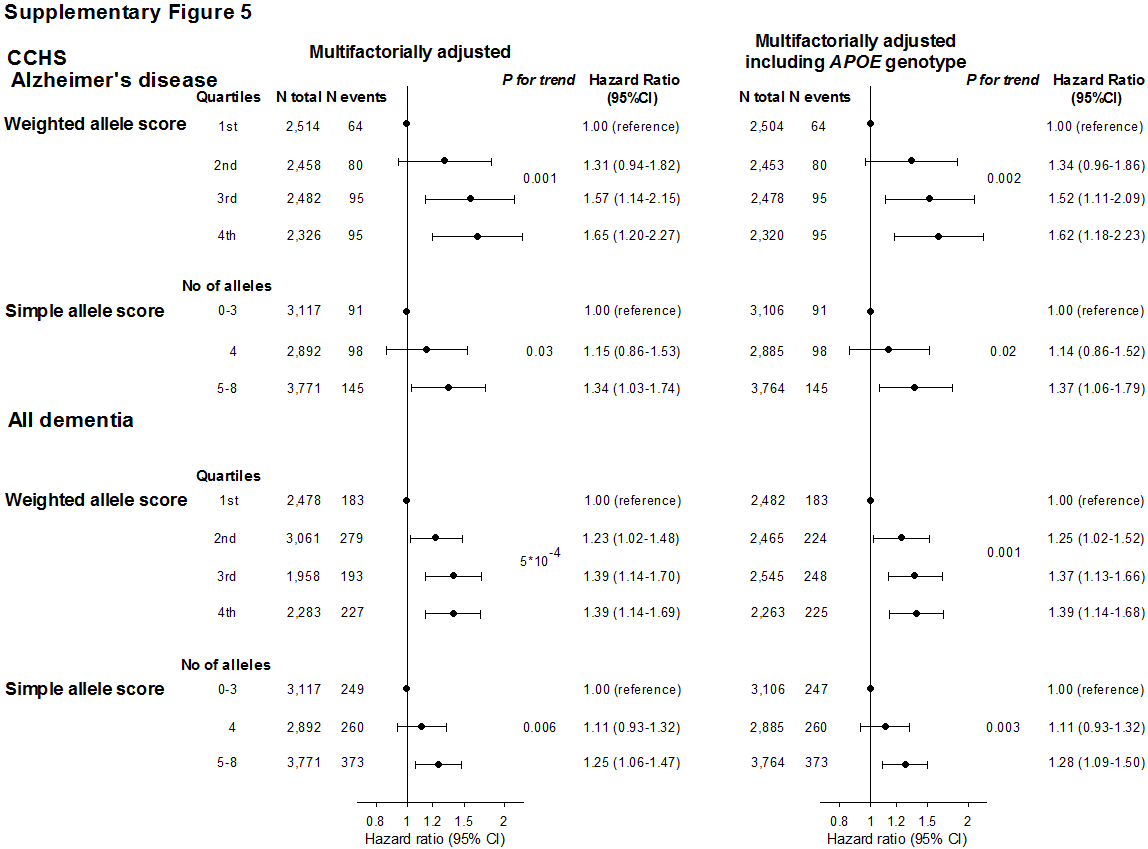


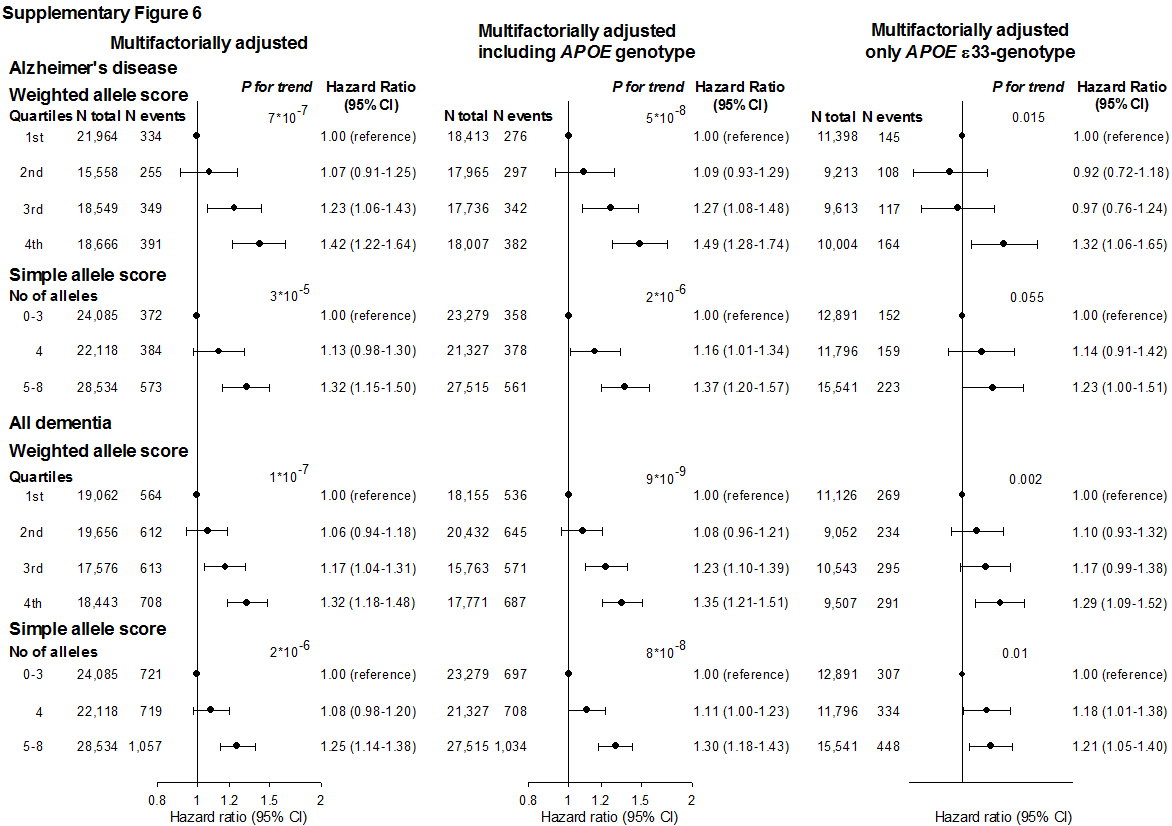


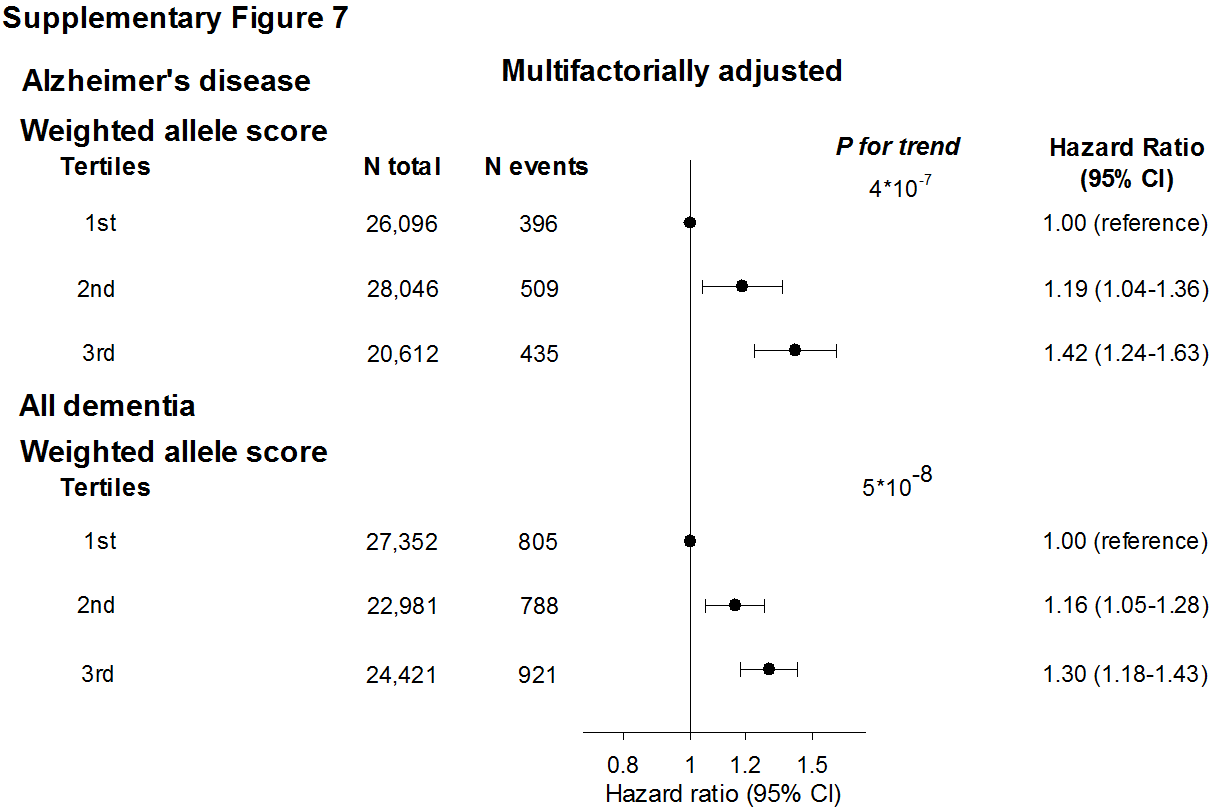


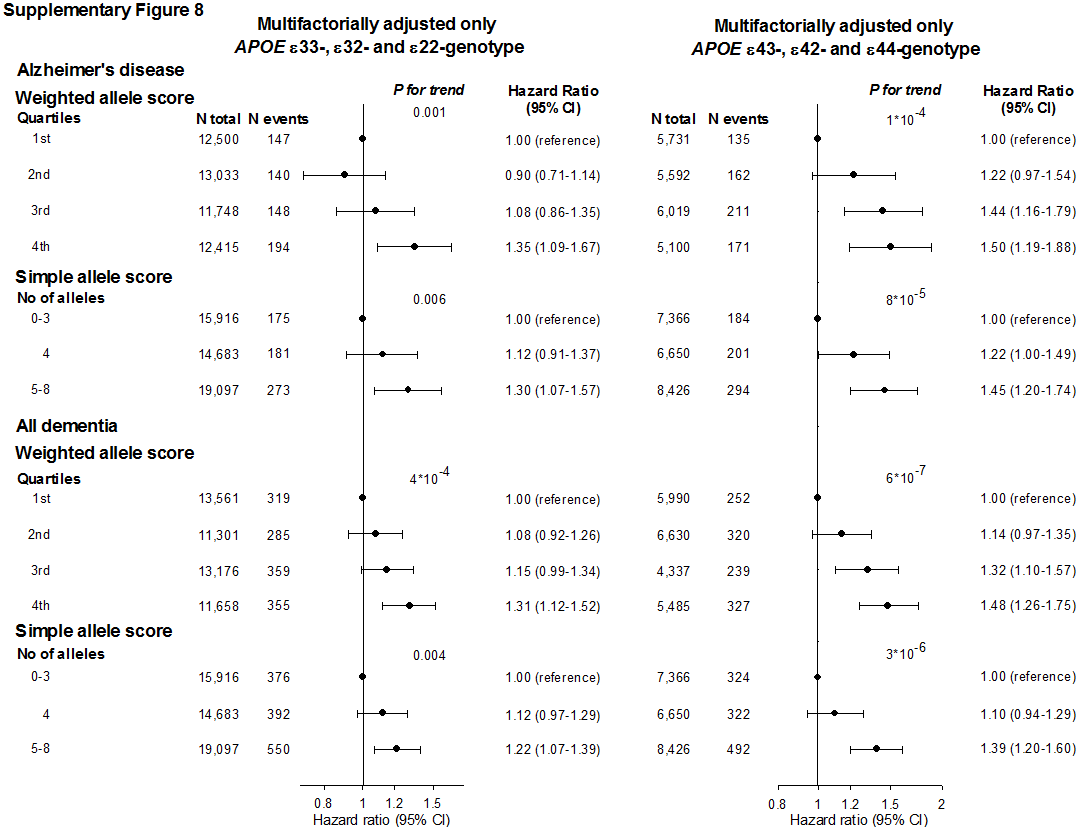


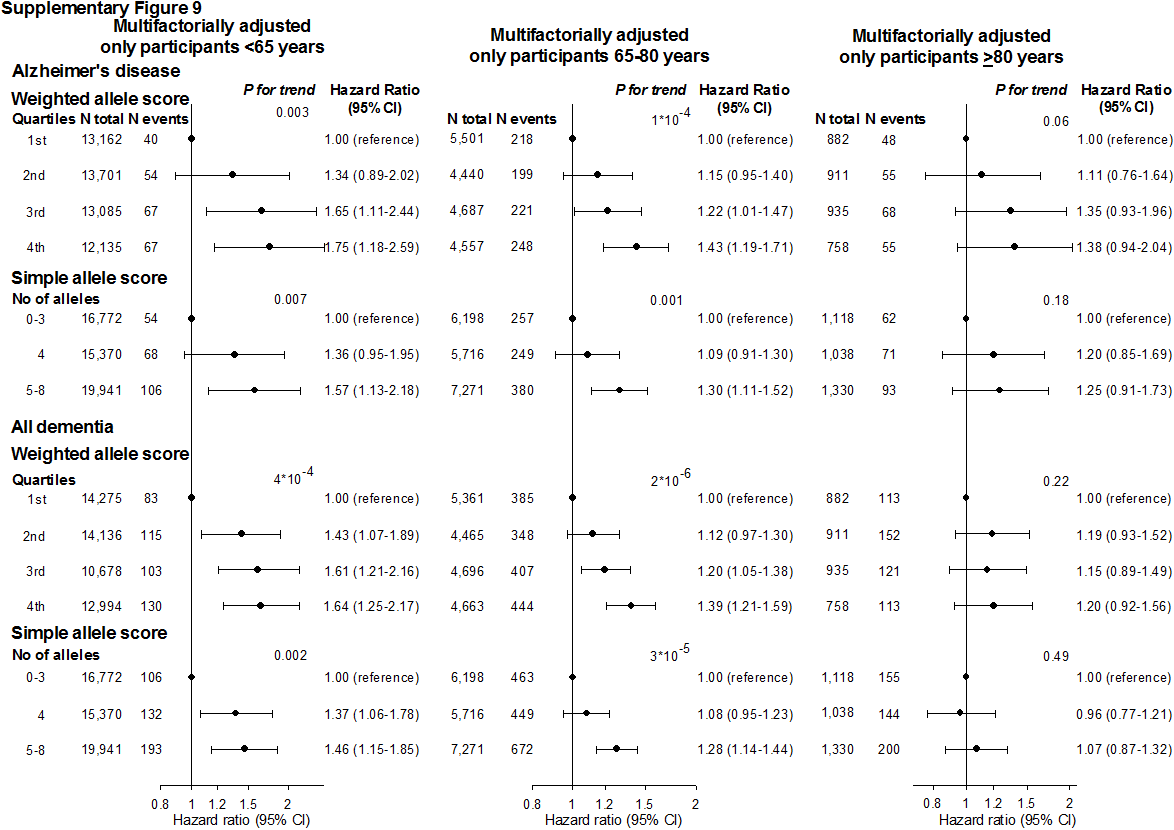


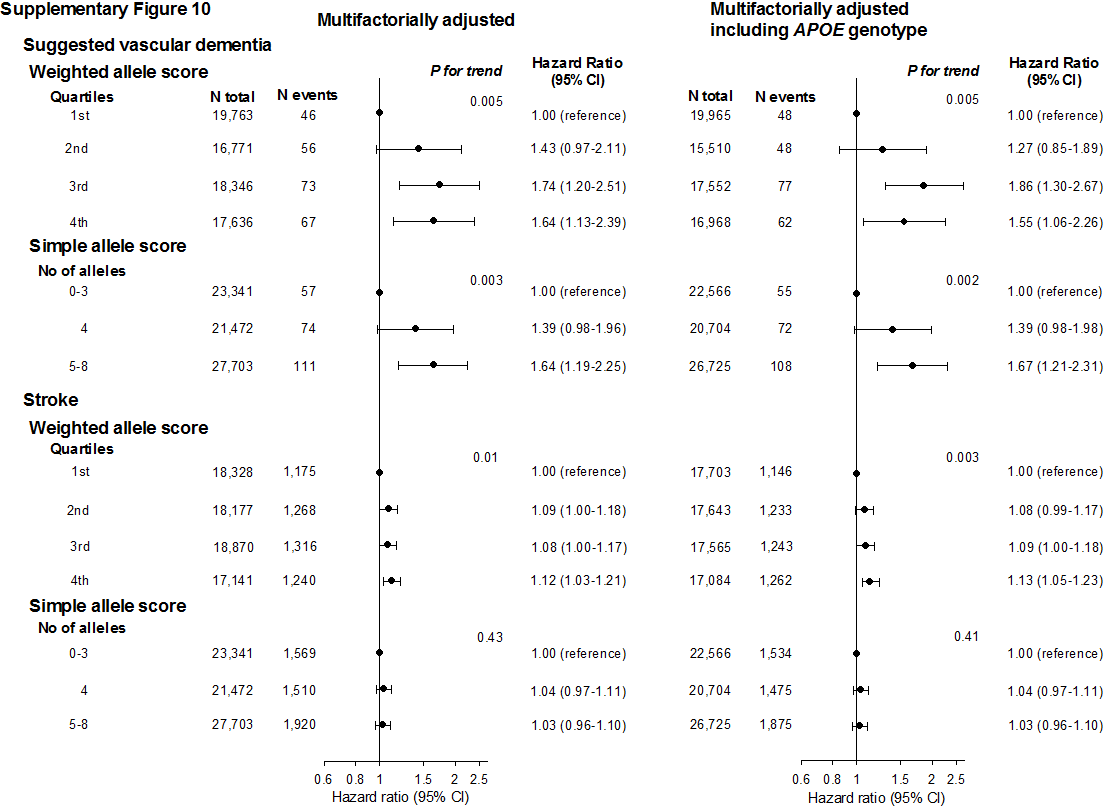

Supplement: Supplementary file 1 — Supplementary material 1 (DOCX 631 kb) [file 10654_2019_498_MOESM1_ESM.docx]
